# Black Gold in Medicine: Rediscovering the Pharmacological Potential

**DOI:** 10.3390/molecules31030408

**Published:** 2026-01-25

**Authors:** Ulduz Hashimova, Aliya Gaisina, Khatira Safikhanova

**Affiliations:** Institute of Physiology Named After Academician Abdulla Garayev, Ministry of Science and Education, AZ1100 Baku, Azerbaijan; aliyagaisina@hotmail.com (A.G.); khatira.safikhanova.74@mail.ru (K.S.)

**Keywords:** crude oil, Naftalan crude, petroleum-derived scaffolds, petroleum biomarkers, sp^3^ scaffolds, petroleomics

## Abstract

This study explores crude oil as a chemically and structurally heterogeneous system with potential pharmaceutical relevance beyond its established roles as an energy and feedstock resource. Recent advances in analytical technologies have enabled the detailed characterization of crude oil constituents at the molecular level, thereby linking structural features to physicochemical properties and possible biological activities. The presented analysis outlines the rationale, methodological considerations, and future research directions for integrating crude oil molecular motifs into the pharmaceutical chemical space. Beyond its conventional role as an industrial and energy resource, crude oil may also hold promise for drug discovery. This study seeks to provide a conceptual framework for reconsidering crude oil as a reservoir of pharmacologically relevant scaffolds and to outline methodological approaches for their systematic assessment. Its rigid sp^3^-rich frameworks, together with sterane/hopane biomarkers, porphyrins, and functional aromatics, structurally overlap with established therapeutic classes and are naturally present in crude oil in suitable abundance, offering opportunities to reduce synthetic effort and expand the chemical space accessible to drug discovery. Advances in petroleomics and in silico methodologies now enable petroleum-derived constituents to be characterized in terms of drug-likeness, bioactivity, and toxicity, providing a framework to reconsider crude oil as an unconventional but analytically and computationally tractable resource for pharmaceutical research.

## 1. Introduction

The pharmaceutical industry is undergoing a profound strategic transformation. Tightening regulatory requirements for evidence-based efficacy of new products, evolving healthcare business models, and the surge of personalized medicine call for a fundamental rethinking of the drug development paradigm from molecular design to real-world applications. Amid global demographic transition, including population aging, and the growing burden of chronic diseases, pharmaceutical companies are being forced to diversify both their R&D portfolios and feedstock strategies, accelerating the shift toward alternative platforms for therapeutic approaches to address long-term, complex healthcare demands [1,2,3,4].

For decades, conventional “drug-likeness” filters—such as Lipinski’s Rule of Five, lead-like, and fragment-based criteria—have constrained the accessible chemical space, excluding many promising scaffolds due to solubility, permeability, or molecular weight cut-offs [5,6,7]. The predominant use of cross-coupling reactions such as Suzuki–Miyaura, and amide formation has historically biased drug libraries toward flat, aromatic scaffolds, without implying intrinsic toxicity or reduced pharmacological potential [8]. Alongside other biological, translational, and clinical challenges, these practices have been recognized as contributing to the attrition observed between preclinical and clinical stages of drug development, which is as high as ~90%, according to Waring et al. (2015) and Sun et al. (2022) [9,10]. Against this backdrop, concepts such as “escape from flatland” (greater sp^3^/3D character), metabolite-likeness, and endogenite orientation have been proposed in the academic literature as alternative design paradigms, accompanied by renewed interest in natural sources of bioactive compounds as drivers of novel therapeutic modalities that meet modern expectations for sustainability, safety, and efficacy [11,12,13,14].

Within this rationale, petroleum—traditionally viewed only as an industrial and energy resource—emerges as a vast, underexplored reservoir of rigid, sp^3^-rich scaffolds and biogenic derivatives for drug design, that do not constitute pharmacophores per se but instead serve as structural starting points for subsequent medicinal chemistry elaboration- an idea that underpins this study.

## 2. Petroleum in Medicine: Practical Insights

Petroleum (crude oil, crudes) is a multicomponent system, generated through microbial and thermocatalytic conversion of ancient organic matter (algae, bacteria, higher plants, plankton, microbial biomass, etc.). During the early stages of petroleum formation (diagenesis), under anoxic depositional conditions, anaerobic microorganisms predominantly mineralize protein and carbohydrate components, releasing O, N, and S as CO_2_, NH_4_^+^, H_2_S, and CH_4_. Lipid and aromatic frameworks are preferentially preserved and transformed, leading to the formation of kerogen—a high-molecular-weight residue of organic matter. In the subsequent stage (catagenesis), as burial continues, within the so-called “oil window” (~60–120 °C, 2–5 km depth) and under increasing pressure, kerogen is transformed into a hydrocarbon-dominated mixture characterized by the prevalence of C–C/C–H skeletons. In practical petroleum chemistry, the vast diversity of components has been summarized by the SARA taxonomy—saturates (S), aromatics (A), resins (R), and asphaltenes (A). The composition of petroleum is determined by the nature of the organic precursor (marine algae, terrestrial plants, microbial biomass), depositional setting (marine vs. lacustrine; carbonate vs. siliciclastic), inorganic input from sediments and formation waters, and the degree of thermal maturity [15]. Under such harsh geological conditions, labile functional groups and reactive side chains are progressively eliminated, yielding relatively thermodynamically stable hydrocarbon structures. Yet, several fundamental molecular backbones—such as steroidal, hopanoid, and porphyrin structures—are selectively retained and serve as biomarkers of petroleum origin and transformation processes and are widely applied in crude oil fingerprinting [16].

In fact, the pharmaceutical and petroleum industries have a long history of operational synergy through established production chains. While the pharmaceutical sector only consumes about 3% of the oil extracted globally, this amount covers almost 99% of its feedstock demand, including aromatics, C_2_–C_3_ olefins/methanol for API synthesis, and polymers (PEG, polypropylene, PVC) for excipients and packaging [17].

To date, the industrial–utilitarian narrative has overshadowed petroleum’s centuries-old role in traditional medicine. Ancient medical sources—from Mesopotamia and Rome to the medieval Middle East and Europe—contain numerous references to how petroleum was esteemed as a natural remedy long before it became a pillar of the energy sector [see Appendix A]. The medicinal use of crude oil was geographically clustered around natural seeps and, although mentioned only intermittently, was included in authoritative pharmacopeias, while its industrial and energy value ultimately came to dominate on a global scale [18,19,20,21,22,23,24,25,26,27,28,29,30,31,32]. Through this lens, petroleum has largely been defined by environmental discourse, which has limited evidence-based evaluation of its potential for drug discovery.

Naftalan is a rare exception. This crude oil deposit near the Naftalan settlement, Azerbaijan, has long been known as a petroleum-based healing site, a tradition also noted in the medieval travel account of Marco Polo [30]. Initially integrated into official sanatorium/rehabilitation medicine practices in the former USSR and later adopted in Croatia, Naftalan’s therapeutic properties have prompted the development of medical preparations, and it is now re-emerging as an empirical platform for early-stage drug discovery [33,34,35,36].

With a century-long history of therapeutic applications across various modalities—such as ointments, cosmetic products, therapeutic baths, or other topical procedures—a substantial body of clinical evidence has been accumulated to empirically validate the efficacy of Naftalan crude oil in the management of complex, chronic conditions such as inflammatory skin diseases (e.g., psoriasis and eczema), autoimmune and degenerative joint disorders (e.g., rheumatoid arthritis and osteoarthritis), neuropathic syndromes (e.g., polyneuropathies and post-injury pain), and vascular complications of metabolic disease [35,36,37,38,39,40].

The ability of Naftalan crude oil to target multiple pathophysiological pathways—including nociceptive signaling, inflammatory cascades, neurodegenerative processes, and endothelial function—is inherently connected to crude oil’s diverse organic makeup and is increasingly viewed as a legitimate asset for pharmaceutical R&D, akin to plant-derived alkaloids or marine biopolymers that have driven modern drug development [41,42].

Efforts have been made to understand the mechanisms behind crude oil-based therapies but were largely constrained by the analytical tools available at the time. Numerous experimental studies were conducted in the Soviet Union and later in post-Soviet countries, but most were published locally and remained largely inaccessible to the international community. Among the most conceptually ambitious was the so-called “precursor theory,” proposed by Yusif Mamedaliyev in the early 1940s and still referenced today. This theory suggests that steranes present in crude oil could be metabolized into biologically active compounds within the human body [43]. Although intellectually compelling, the hypothesis lacked experimental validation, primarily because technologies capable of tracing such biochemical transformations at the molecular level did not yet exist. However, this remains a critical issue for the pharmaceutical sector: elucidating the metabolic fate of crude oil constituents is a prerequisite for assessing both their therapeutic potential and safety.

Today, advanced analytical chemistry techniques allow for in-depth compositional analysis, including heavy petroleum fractions, serving as a critical first step in identifying previously inaccessible molecular structures that may hold significant pharmaceutical potential. When integrated into the traditional drug discovery pipeline—from computational modeling and virtual screening to in vitro and in vivo evaluation—these methods enable systematic exploration of bioactivity profiles, accelerating the identification of drug-like candidates within complex hydrocarbon matrices.

## 3. Pharma Matrix of Crude Oil: Structural Insights

Naftalan crude oil represents a distinctive class of biodegraded heavy crudes, with physicochemical properties setting it apart from conventional fuel-grade petroleum. It is characterized by high density (0.910–0.960 g/cm^3^), extreme viscosity, and, most notably, a near-total absence of alkanes. In their place, it contains an unusually high proportion of naphthenic hydrocarbons—approximately 59.4–77.0% depending on the stratigraphic horizon and processing method—and remarkable enrichment in decahydronaphthalenes, as evidenced by a dominant diagnostic fragment ion at *m*/*z* 95 (mass-to-charge ratio), characteristic of saturated bicyclic naphthenic hydrocarbons. In addition, Naftalan oil shows elevated levels of trace elements (Ba, Fe, Ni, Ti, and Zn) and even noble metals (Au, Pd, Pt, Rh, and Te) [41,44,45].

Critically, such specific chemical profile grants access to a variety of non-flat, sp^3^-rich rigid hydrocarbon frameworks—from simple mono- and bicyclic rings to more elaborate fused or bridged polycycles—that were long overlooked but are now emerging as a new trend in drug discovery scaffold design. This paradigm shift, often described as the “escape from flatland”, emphasizes the transition from flat aromatic systems to three-dimensional architectures [11,12]. Sp^3^-rich frameworks provide structural and functional advantages: they improve shape complementarity with hydrophobic protein cavities, enhance metabolic stability, and can increase lipophilicity, facilitating membrane permeability. In contrast, planar aromatic systems are intrinsically rigid and conformationally restricted, often prone to rapid metabolic oxidation and associated with genotoxic risks due to their ability to intercalate with nucleic acids. Moreover, the sp^3^-rich frameworks present in petroleum frequently retain stereogenic centers inherited from their biological precursors—steranes (from sterols), hopanes (from bacterial triterpenoids), chiral isoprenoids such as pristane and phytane, and a range of diterpenoid and triterpenoid derivatives—thereby introducing chirality that enhances selective interactions with biological targets. Together, these features provide advantages over planar plant-derived alkaloid frameworks and position them as ready-made bioisosteric replacements for classical functional groups [46,47].

**Figure 1:**
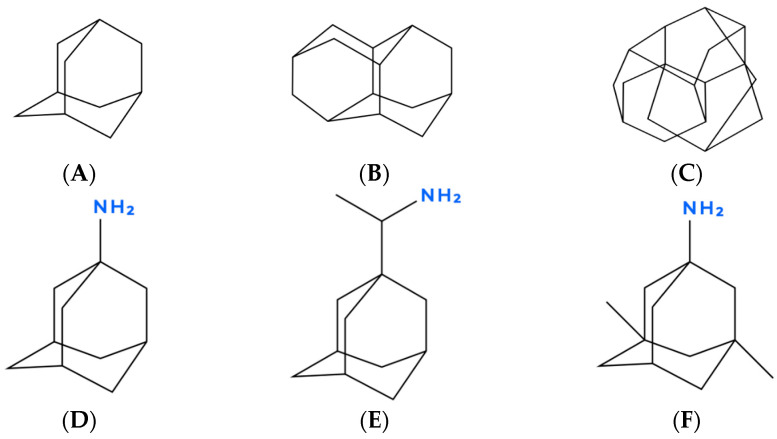
Schematic representations of the adamantane cage scaffold (**A**) and higher diamondoids—diamantane (**B**) and triamantane (**C**)—illustrating rigid sp^3^-hybridized cage architectures. Clinically used adamantane derivatives are shown in (**D**–**F**): amantadine (**D**), rimantadine (**E**), and memantine (**F**).

The most widely recognized and clinically validated petroleum-derived scaffolds are adamantanes, also known as diamondoids (see Figure 1). They are fully sp^3^-hybridized hydrocarbon cages based on the adamantane framework (tricyclodecane, C_10_H_16_) that have a diamond-lattice-like carbon skeleton and exceptional stiffness and rigidity. First identified by Mobil Oil Corporation as problematic deposits clogging pipelines, diamondoids were later recognized as valuable molecular building blocks for pharmacologically active compounds [48,49]. Structural features such as high symmetry (Td), complete saturation, and compact 3D cage architecture account for their effectiveness in pharmacology and medicine—either as the so-called “lipophilic bullet,” providing critical lipophilicity when appended to known pharmacophores, or through intrinsic activity linked to the modulation of ion channels, in particular NMDA receptors and viral proton channels [50]. The rigid lipophilic cage further enhances membrane permeability and metabolic stability and may preferentially partition into lipid rafts, thereby influencing membrane organization and associated signaling pathways. Adamantane itself has become the most extensively studied and clinically validated scaffold, forming the structural core of approved drugs such as rimantadine (antiviral), amantadine (anti-Parkinson’s), and memantine (anti-Alzheimer’s) [50,51]. More complex members of this family and their derivatives (amines, carboxylic acids, esters, and anilines) have been investigated and show promising antiviral, anticancer, and neuroprotective activities, although none have yet reached clinical applications [48,49]. Importantly, petroleum and natural gas condensates contain an even richer diversity of higher diamondoids, most of which remain unexplored as potential scaffolds for future drug discovery.

**Figure 2:**
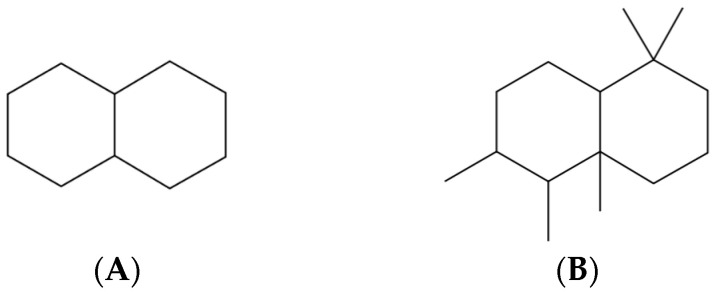
Bicyclic scaffolds: decalin (**A**) and drimane (**B**) schematic representations.

Decalins (decahydronaphthalenes, C_10_H_18_) are simple saturated bicyclic hydrocarbons composed of two fused cyclohexane rings in cis- or trans-configuration (see Figure 2A). They represent one of the most fundamental rigid sp^3^ scaffolds, occurring both as synthetic models and as structural motifs in a wide range of bioactive terpenoids and steroids [52]. In petroleum, decalins are characteristic constituents of naphthenic crudes, such as Naftalan oil (Azerbaijan), where decahydronaphthalenes account for up to ~60% of the hydrocarbon fraction, making them a structural hallmark of this crude. While petroleum-derived decalins themselves have not yet been systematically investigated in clinical medicine, decalin motifs are widely represented in natural products and synthetic scaffolds of pharmacological relevance. Notable examples include decalin–tetramic acid hybrids, such as equisetin and zopfiellamides, which have broad-spectrum antibacterial and antifungal activity; macrolides, such as nodusmicin, which are effective against drug-resistant pathogens; and synthetic decalin-based scaffolds for FKBP51 inhibitors, which are currently under investigation as potential treatments for depression, obesity, and cancer [53].

Drimanes are a family of bicyclic sesquiterpenes (C_15_H_28_) based on a trans-decalin core, with homo-drimanes representing extended variants of this skeleton (see Figure 2B). First isolated as drimenol from *Drimys winterii*, these frameworks are widespread in plants, fungi, and marine organisms and were also detected in petroleum as early as the 1970s [54]. They are thought to originate from the degradation of higher plant triterpenes or bacteriohopanoids during diagenesis, giving rise to characteristic 8β(H)-drimane and homodrimane isomers. Pharmacologically, drimane-type sesquiterpenoids have demonstrated a broad spectrum of experimental activities, including anticancer, anti-inflammatory, antiviral, antifungal, neurotrophic, and enzyme-inhibitory effects [55]. Although diverse drimane and homo-drimane derivatives have been identified in crude oils, their evaluation as scaffolds for drug discovery remain limited.

**Figure 3:**
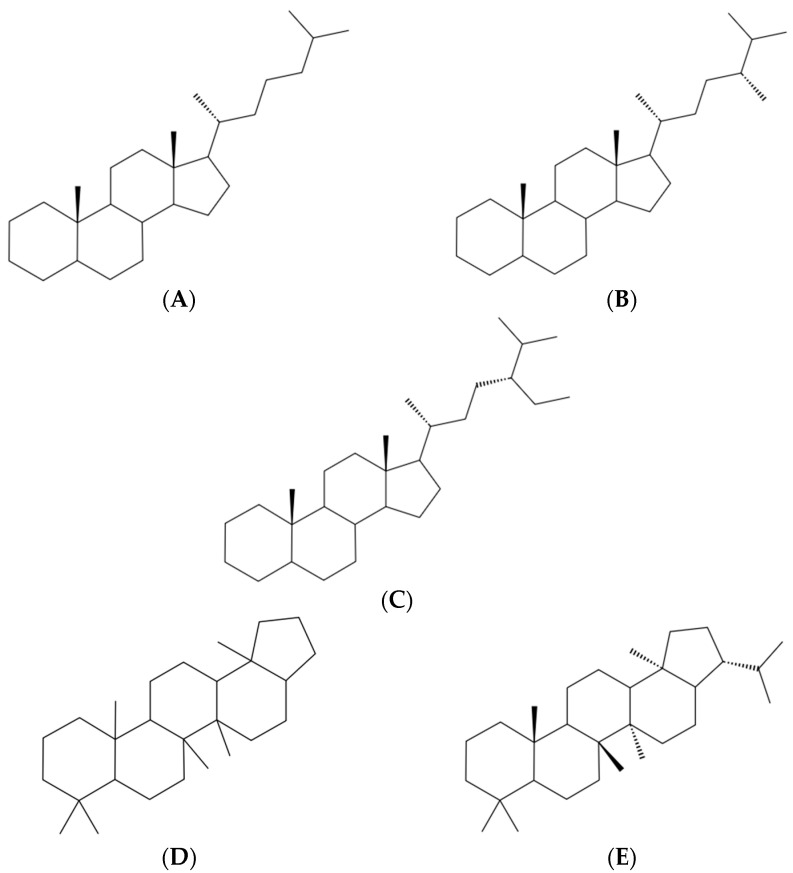
Schematic representations of petroleum-derived polycyclic biomarkers, illustrating two distinct core architectures: steranes, built on the tetracyclic cyclopenta-perhydrophenanthrene (gonane) nucleus (**A**–**C**; cholestane-, ergostane-, and stigmastane-type structures), and hopanes, derived from pentacyclic bacterial hopanoid triterpenes (**D**,**E**).

Steranes and hopanes are polycyclic hydrocarbons derived from steroids and pentacyclic triterpenes during diagenesis and catagenesis. Steranes contain the tetracyclic steroid nucleus (gonane, cyclopentanoperhydrophenanthrene), directly homologous to the core of cholesterol and human steroid hormones (e.g., androstane-, estrane-, and cholestane-type derivatives), and they are intrinsically chiral, with multiple stereogenic centers controlling biological specificity. In contrast, hopanes possess the pentacyclic skeleton derived from bacterial hopanoids, reflecting prokaryotic membrane architecture (see Figure 3).

Both classes are essential to membrane organization, contributing to the formation and stabilization of lipid rafts—nanodomains that modulate receptor clustering, signal transduction, and viral entry—so petroleum-derived sterane and hopane frameworks may provide a basis for biomedical applications targeting membrane-associated signaling [56,57,58,59]. Traditionally considered solely as geochemical biomarkers, these compounds have only recently been evaluated for their pharmacological potential, with Naftalan oil representing one of the first systematic case studies. Calculated reactivity descriptors and PASS (Prediction of Activity Spectra for Substances)-based screening predicted anti-inflammatory, antimicrobial, antiviral, hepatoprotective, immunomodulatory, and antitumor activities, while QSAR (Quantitative Structure–Activity Relationship) models suggested low to moderate toxicity (LD_50_ ≈ 750–1400 mg/kg). This integrated virtual pipeline provides a rationale for prioritizing petroleum-derived biomarkers as candidate scaffolds [60].

Heavy crude oils may host nanostructures of pharmaceutical relevance. In 2020, researchers at the Max Planck Institute for Coal Research (MPI für Kohlenforschung, Mülheim) reported the presence of a broad spectrum of fullerenes in the asphaltene fraction of heavy oil, including classical buckminsterfullerenes and buckybowls—hemispherical aromatic structures with unique reactivity and potential applications in biomedicine, catalysis, and drug development [61].

**Figure 4:**
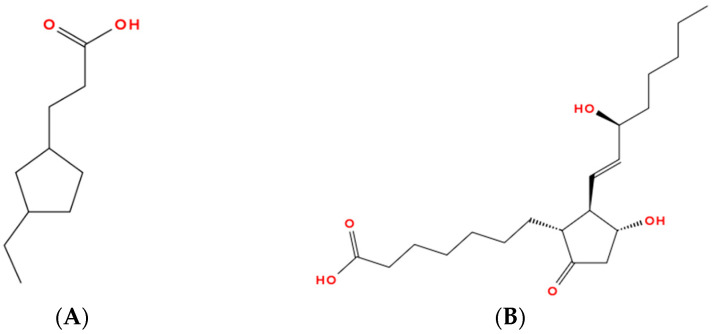
Representative structure of a naphthenic acid (**A**) shown in comparison with prostaglandin E_2_ (**B**), a canonical cycloaliphatic lipid mediator involved in inflammatory signaling, to illustrate potential structural similarities.

Naphthenic acids—mixtures of cycloaliphatic carboxylic acids abundant in naphthenic crudes such as Naftalan—combine lipophilicity and polarity through sp^3^-rich cyclic cores and a carboxyl group. Their structural analogy to prostaglandins suggests that they have possible roles as bioisosteres in inflammatory and age-related processes, though biomedical data remain limited and at times contradictory (see Figure 4) [62].

**Figure 5:**
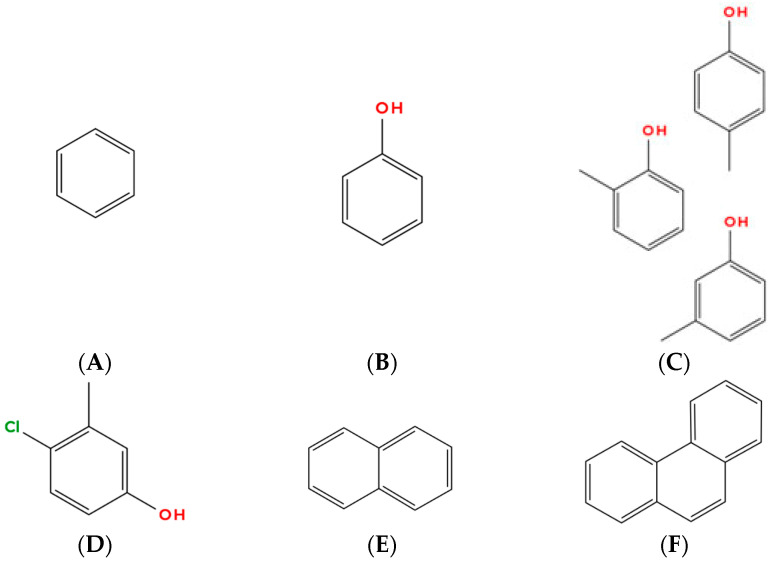
Schematic representations of aromatic, substituted aromatic and polyaromatic frameworks relevant to petroleum, including benzene (**A**), phenol (**B**), cresols (**C**), chlorocresol (**D**), naphthalene (**E**), and phenanthrene (**F**).

**Aromatic hydrocarbons**—planar conjugated π-systems ranging from simple benzenes to polycyclic aromatic hydrocarbons (PAHs)—are ubiquitous in petroleum, where low-molecular-weight compounds (phenol, cresols, and chlorocresols) constitute an important feedstock for medicinal chemistry (see Figure 5). By contrast, polycyclic fractions within asphaltenes are generally regarded as waste and a source of environmental concern. Pharmacologically, aromatic scaffolds engage targets via π–π stacking with aromatic residues and hydrophobic interactions in receptor binding sites; heteroaromatics add hydrogen-bonding capacity and electronic modulation. Notably, extended PAHs can intercalate into DNA, a mechanism underlying the activity of anthracyclines and related agents but also their genotoxic potential. In addition, PAHs partition into lipid bilayers, where they may influence membrane fluidity, lipid raft stability, and receptor clustering. From a druggability perspective, however, an increasing aromatic ring count correlates negatively with oral bioavailability and aqueous solubility and raises safety risks through high plasma–protein binding and CYP/hERG liabilities [63]. Even so, heteroaromatic and fused aromatic systems remain foundational in drug discovery—forming the backbone of many antibiotics, antivirals, antipsychotics, and anticancer agents—provided planarity and polarity are carefully optimized [4,64,65,66,67].

**Figure 6:**
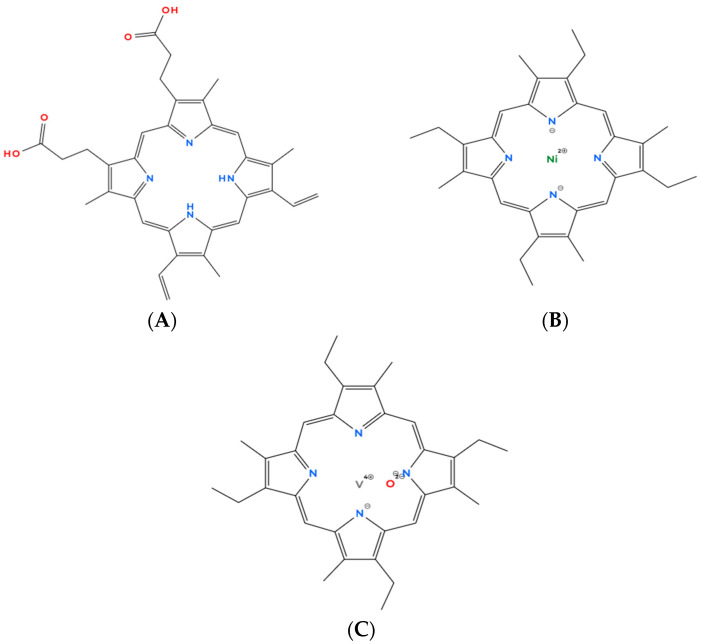
Representative tetrapyrrolic scaffolds illustrating the transition from biological to petroleum-derived porphyrins: protoporphyrin IX (**A**), nickel etioporphyrin (**B**), and vanadium (IV) etioporphyrin III oxide (**C**). The figure highlights preservation of the porphyrin macrocycle during diagenesis and metal complexation in crude oil.

Petroporphyrins—naturally occurring metalloporphyrins in crude oil—are structurally related to biological tetrapyrroles such as heme and chlorophyll [68]. These macrocyclic compounds, primarily nickel and vanadium complexes, were first identified in petroleum in the 1930s. Like their biological counterparts, petroporphyrins are essentially planar, a conformation that supports π-conjugation, metal coordination, and characteristic optical properties (see Figure 6). Their porphyrin scaffold includes chemically accessible meso- and β-positions, making them promising substrates for structural modification. Recent structural studies (UV–vis, FT-ICR MS, and AFM) have shown that petroporphyrins preserve their geometric substitution patterns even in more complex forms, highlighting their stability and potential as modifiable scaffolds [69]. While porphyrin derivatives are already approved for photodynamic therapy (e.g., temoporfin and verteporfin) and are being explored as antioxidants, radioprotectors, and antimicrobial agents, petroleum-derived porphyrins remain an underexplored resource in pharmaceutical chemistry despite their ready availability and inherent structural diversity [70,71,72,73].

Organosulfur compounds are abundant in petroleum and generally seen as toxic, but Ichthyol (ammonium bituminosulfonates), derived from sulfur-rich shale oil, represents a notable exception. Used in dermatology for over a century, it demonstrates antiseptic, anti-inflammatory, and immunomodulatory effects, now supported by modern studies confirming activity even against resistant strains of *Staphylococcus aureus* [74,75].

## 4. Translational Perspectives of Crude Oil in Drug Discovery

The data reviewed in this article emphasize that crude oil is more than a petrochemical feedstock: it can be regarded as a structurally intricate mixture comprising molecular motifs that range from flat aromatic units to rigid, three-dimensional architectures. Many of these structures exhibit drug-like features and may serve as templates for medicinal chemistry and rational drug design, thus providing new translational opportunities. Identified biological activities of petroleum-derived scaffolds align with pressing unmet medical needs in different fields, including oncology (over 30% of global R&D efforts), cardiometabolic disorders, antimicrobial and immunomodulatory therapies, and the fast-growing neuroscience field addressing neurodegeneration and psychiatric conditions [4]. Advances in chemical biology and in silico technologies now enable these components to be positioned not merely as generic leads but as sources of first-in-class molecules with novel mechanisms of action, a highly relevant paradigm given the stagnation of conventional drug discovery [76]. Importantly, biomarkers from Naftalan oil show predicted low toxicity and multi-target potential, a strategy increasingly adopted by the pharmaceutical industry to address multifactorial diseases and reduce polypharmacy [77,78].

Moving from concept to practical application will require considerable multidisciplinary effort, integrating expertise from chemistry, pharmacology, toxicology, and computational sciences. Unlike botanical or microbial natural product databases, there is no integrated pharmacological resource covering oil-derived molecules. Petroleomics—ultrahigh-resolution mass spectrometry of crude oils—has revealed thousands of individual components (≥3000 identified, potentially tens of thousands with isomers) across C_10_–C_>50_ hydrocarbons and heteroatom-containing derivatives, with dedicated workflows for classification [79,80].

However, while such approaches have established powerful databases for geochemistry and ecotoxicology, they are largely disconnected from pharmacological contexts. A translational framework would require systematic annotation of descriptors—including sp^3^ fraction, lipophilicity, polar surface area, chirality, and quantum chemical reactivity indices—to transform petroleomic fingerprints into pharmacologically meaningful data streams [81,82]. Once such datasets are established, they could be fed into the classical drug discovery pipeline starting with in silico approaches (virtual screening, molecular docking, QSAR modeling, and structure-based optimization), extending to in vitro functional assays (receptor binding and enzymatic and cell-based systems), and culminating in in vivo validation studies to assess pharmacokinetics, efficacy, and safety.

Although this strategy offers a pathway for incorporating petroleum into pharmaceutical R&D, significant challenges remain—and many are not entirely unique to petroleum. Natural products from plants and microbes likewise present issues of chemical complexity, inherent toxicity, and the need for scalable purification [83,84]. Crude oil, however, is an extraordinarily complex mixture, with many constituents occurring as unresolved isomeric ensembles, complicating isolation and structural elucidation. Certain fractions, such as PAHs and naphthenic acids, are well known for their toxicity and ecotoxicological impact, underscoring the need for early toxicity prediction and rigorous filtering before pharmacological exploration. Technical barriers also persist, and reproducible separation methods, scalable purification strategies, and integration of advanced analytics with machine learning will be essential to navigate this chemical complexity.

## 5. Conclusions

The medicinal use of petroleum is not a new or regionally confined concept: crude oil derivatives have occasionally entered medical practice, often through the repurposing of by-products. The evidence reviewed in this study demonstrates that crude oil harbors a broad spectrum of biologically relevant scaffolds, ranging from rigid sp^3^-rich frameworks such as adamantanes, steranes, and hopanes to planar π-systems including porphyrins and functional aromatics, several of which structurally overlap with established therapeutic classes, without implying direct pharmacophoricity. Importantly, many of these motifs—some of which are unique to petroleum or occur at the industrial scale—offer an opportunity to potentially reduce synthetic effort and expand the chemical space accessible to drug discovery. Yet, petroleum-derived pharmacology is in its infancy and not a clearly defined field: while petroleomics has revealed extraordinary molecular diversity for geochemical purposes, its integration into drug discovery remains limited. Bridging this gap—through safety assessment, biological profiling, and cheminformatics-driven scaffold design—may catalyze the emergence of a distinct research direction in pharmacology.

## Data Availability

All data used in this article are available within the main text. Further inquiries should be directed to the corresponding author.

## Appendix A

### Historical Timeline of the Medical Use of Petroleum

Long before petroleum became an industrial resource, it was a significant component of medical and ritual practices in regions where natural seepages of crude oil or bitumen occurred. Without attempting to be exhaustive, the timeline below captures key historical moments when such empirical practices were translated into documented medical knowledge.

I. Mesopotamia and the Ancient Near East (ca. 1st millennium BCE).

The earliest documented evidence of medicinal petroleum use derives from cuneiform texts of Assyrian and Babylonian origin, including tablets listed in the Aššur Medical Catalogue (AMC) and therapeutic prescriptions preserved in the British Museum corpus (*Cuneiform Texts from Babylonian Tablets*). These sources refer to *napṭu*, a naturally seeping flammable substance, primarily applied externally for the treatment of wounds, skin diseases, and for ritual cleansing [18,19]. Mesopotamian accounts of hydrocarbon substances—particularly *napṭu* (liquid petroleum) and *kupru* (bitumen/asphalt)—were echoed both linguistically and empirically in later Greek notions of *naphtha* and Roman descriptions of petroleum use.

II. Classical Antiquity (5th century BCE–5th century CE).

In Greco-Roman medicine, petroleum entered the formal canon of *materia medica*. References appear in the *Corpus Hippocraticum*, while Dioscorides (*De Materia Medica*, Book I) described naphtha and bitumen as warming and resolvent agents [20]. Pliny the Elder (*Naturalis Historia*, Books II and XXXV) documented geographic sources and therapeutic applications of petroleum and asphalt [21]. Within Galenic humoral theory, bituminous substances such as asphaltum and naphtha were classified as “hot and dry” and were recommended primarily for external therapeutic use [22].

III. Arabic and Persian Medicine (9th–13th centuries).

Medieval Islamic medical literature provides the most systematic pharmacological descriptions of petroleum (*naft*) use. Avicenna devoted a specific section to *naft* in *al-Qānūn fī al-Ṭibb*, distinguishing several types and recommending primarily external use for joint pain, inflammation, parasitic infestations, and skin diseases [23]. Muḥammad ibn Zakariyyāʾ al-Rāzī discussed *naft* in clinical contexts in *Kitāb al-Ḥāwī fī al-Ṭibb* (*Liber Continens*) [24]. Serapion the Elder (Yūḥannā ibn Sarābiyūn) included petroleum in his pharmacological compendium known in Latin as *Liber Aggregatus in Medicinis Simplicibus* [25]. Ibn al-Bayṭār, in *Kitāb al-Jāmiʿ li-Mufradāt al-Adwiya wa-l-Aghdhiya*, compiled and systematized earlier Greek and Arabic knowledge on petroleum [26].

IV. Medieval and Early Modern Europe (11th–18th centuries).

In the 11th century, Constantine the African translated Arabic medical texts into Latin, thereby introducing references to petroleum into European medical literature, particularly through the Salernitan medical school [27]. Crude oil was subsequently included in medieval pharmacopeias, appearing in the *Antidotarium Nicolai* (late 11th–12th century), one of the most authoritative medical formularies of its time, and later in works by Roger of Parma, Gilbertus Anglicus (*Compendium Medicinae*, c. 1230–1250), and Matteo Silvaticus (*Pandectae Medicinae*, early 14th century) [25].

From the late fifteenth century onward, this body of knowledge continued to circulate through printed pharmacological compendia—such as the French *Arbolayre* and *Grant Herbier en françois*—as well as within the German *Hortus Sanitatis* corpus, initiated by Peter Schöffer in Mainz (1485) and further developed in editions by Johann von Cube (1485) and Jacobus Meydenbach (1491) [25].

Additionally, short practical pamphlets were widely published, serving as concise guides to the therapeutic use of petroleum and related substances in routine medical practice [25]. Such treatments relied primarily on petroleum obtained from natural oil seepages, including those of southern France (Font de l’Oli at Gabian, within the Montpellier medical sphere), northern Italy (oil of St Catherine near Modena), eastern France and Alsace (Pechelbronn and Lobsann), northern Germany (Hanover region), and the Carpathian oil-bearing zones of Central Europe historically associated with Galicia and the Austro-Hungarian lands. Contemporary descriptions consistently emphasize the medicinal value of untreated petroleum—dark, viscous, and adhesive—whereas distilled or refined petroleum products were not considered therapeutically equivalent [25]. Bituminous materials were employed in medical and veterinary contexts, as well. In England, surface bitumen, oil-impregnated peat, and heavy tar-like substances derived from shallow seepages were used on a limited and localized scale. In Switzerland, natural asphalt (asphaltum) was identified at Val-de-Travers near Neuchâtel in the early eighteenth century and similarly attracted medical interest, reflecting a broader European engagement with native bituminous substances prior to industrial exploitation [28].

V. Nineteenth Century to Modern Times.

Early mid 19th century (United States). Prior to industrial oil production, naturally occurring petroleum was widely marketed for medicinal purposes. Following the discovery of petroleum sources in 1829, products such as “American Medicinal Oil” (Kentucky) and “Seneca oil” (Lake Seneca, New York) were commercially distributed as external remedies [29]. Between 1846 and 1858, large-scale medicinal trade was conducted by Samuel Martin Kier, who promoted crude oil-based remedies (“Kier’s Genuine Petroleum” or “Rock Oil”) through printed leaflets and nationwide wagon distribution networks, selling approximately 250,000 bottles, preceding the transition to industrial petroleum exploitation in 1859.

Late 19th–20th centuries (Europe). From the late nineteenth century onward, petroleum re-emerged in European medical practice through the Naftalan deposit. In 1892, the German engineer Y. I. Eger initiated systematic drilling and production of Naftalan-based ointments, exported to Europe. Clinical use was documented in 1896 by F. G. Rozenbaum [30], and between 1912 and 1914 Naftalan-based medicines were manufactured in Germany by *Magdeburg-Naftalan* and *Dresden-Naftalan*, with formulations published in *Pharmaceutische Zeitung* (Berlin, 1899) [31]. During the Soviet period, Naftalan was incorporated into official medical practice through the establishment of a specialized sanatorium and formal recognition in 1926. In the late 1960s, a petroleum source with comparable therapeutic properties was identified in Ivanić-Grad (Moslavina region, Croatia), where medical use was institutionalized in the 1980s and continues to the present day.
